# 1727. Phase 1b Dose-ranging Study Demonstrates Tolerability and Pharmacokinetics (PK) of Oral Epetraborole at the Predicted Therapeutic Dosage for *Mycobacterium avium* Complex (MAC) Lung Disease

**DOI:** 10.1093/ofid/ofac492.1357

**Published:** 2022-12-15

**Authors:** Paul B Eckburg, Dave Clarke, Jennifer Long, Sanjay Chanda, Kevin M Krause, Eric Easom, George Talbot, Christopher M Rubino, Angela Molga

**Affiliations:** AN2 Therapeutics, Inc., Menlo Park, CA; AN2 Therapeutics, Inc., Menlo Park, CA; AN2 Therapeutics, Inc., Menlo Park, CA; AN2 Therapeutics, Inc., Menlo Park, CA; AN2 Therapeutics, Inc., Menlo Park, CA; AN2 Therapeutics, Inc., Menlo Park, CA; Talbot Advisors LLC, Bradenton, Florida; Institute for Clinical Pharmacodynamics, Schenectady, New York; Royal Adelaide Hospital, Adelaide, South Australia, Australia

## Abstract

**Background:**

Epetraborole (EBO) — an orally available bacterial leucyl transfer RNA synthetase inhibitor with potent activity against nontuberculous mycobacteria — is under clinical development for treatment of MAC lung disease. We conducted a Phase 1b dose-ranging study of EBO tablets in healthy adult volunteers, to inform dose selection in the treatment of MAC lung disease.

**Methods:**

In this double-blind, placebo-controlled trial, EBO or placebo tablets were administered (n=8/cohort, 3:1 randomization) at dosages of 250-1000 mg q24h or 500 mg or 1000 mg q48h for up to 28 days. Standard Ph1 clinical and laboratory evaluations and treatment-emergent adverse events (TEAEs) were assessed. Based on prior human studies using significantly higher EBO daily doses, gastrointestinal (GI) events and anemia were predetermined AEs of special interest (AESIs). Plasma concentrations of EBO were measured by validated LC-MS/MS methods. Plasma PK parameters were determined using non-compartmental methods.

**Results:**

A total of 43 subjects were enrolled; the 1000 mg q24h cohort was terminated early due to local COVID restrictions. Overall, 80.6% EBO subjects and 83.3% placebo subjects experienced ≥1 TEAE, none of which was serious or severe (Table). Most TEAEs were mild in severity (90%), and the remainder were moderate (10%). No TEAE leading to withdrawal from study was reported. The most frequent types of TEAEs were GI events (48.4% EBO, 41.7% placebo subjects), the most common being mild nausea. Two subjects had premature discontinuation of EBO due to a TEAE (asymptomatic liver enzyme elevations in a 250 mg q24h subject and mild nausea in a 1000 mg q48h subject). One 1000mg q24h subject had a TEAE of anemia. No clinically significant findings or TEAEs were observed for physical examinations, ECGs, or urine laboratory tests. Plasma C_max_ and AUC_0-∞_ of EBO increased in a linear, dose-proportional manner across cohorts. T_max_ was observed at ∼1 h post dose; mean t_1/2_ ranged from 7.63 to 11.1 h.

**Figure:**
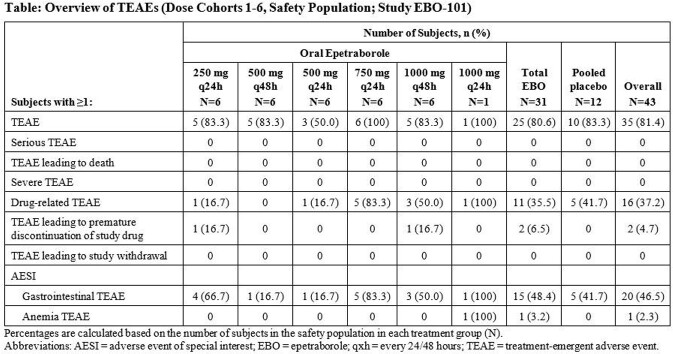

**Conclusion:**

- Oral EBO administered for 28-day dosing was generally well tolerated at the predicted therapeutic dose (500mg q24h)
- Predictable PK characteristics facilitate its use in MAC lung disease
- Further evaluation in a Phase 2/3 treatment-refractory MAC lung disease study is planned

**Disclosures:**

**Paul B. Eckburg, MD**, Paratek Pharmaceuticals, Inc.: Safety Review Board|Spero Therapeutics, Inc.: Advisor/Consultant **Sanjay Chanda, PhD**, AN2 Therapeutics: Stocks/Bonds **George Talbot, MD**, AN2 Therapeutics, Inc.: Advisor/Consultant|AN2 Therapeutics, Inc.: Co-founder **Christopher M. Rubino, PharmD**, Adagio Therapeutics: Grant/Research Support|Amplyx Pharmaceuticals, Inc: Grant/Research Support|AN2 Therapeutics: Grant/Research Support|Antabio SAS: Grant/Research Support|Arcutis Biotherapeutics, Inc: Grant/Research Support|B. Braun Medical Inc.: Grant/Research Support|Basilea Pharmaceutica: Grant/Research Support|Boston Pharmaceuticals: Grant/Research Support|Bravos Biosciences: Ownership Interest|Celdara Medical LLC: Grant/Research Support|Cidara Therapeutics Inc: Grant/Research Support|Cipla USA: Grant/Research Support|Crestone Inc: Grant/Research Support|CXC: Grant/Research Support|Debiopharm International SA: Grant/Research Support|Entasis Therapeutics: Grant/Research Support|Evopoint Biosciences Co.: Grant/Research Support|Fedora Pharmaceuticals: Grant/Research Support|GlaxoSmithKline: Grant/Research Support|Hoffmann-La Roche: Grant/Research Support|ICPD: Ownership Interest|ICPD Biosciences, LLC.: Ownership Interest|Insmed Inc.: Grant/Research Support|Iterum Therapeutics Limited: Grant/Research Support|Kaizen Bioscience, Co.: Grant/Research Support|KBP Biosciences USA: Grant/Research Support|Lassen Therapeutics: Grant/Research Support|Matinas Biopharma: Grant/Research Support|Meiji Seika Pharma Co., Ltd.: Grant/Research Support|Melinta Therapeutics: Grant/Research Support|Menarini Ricerche S.p.A: Grant/Research Support|Mutabilis: Grant/Research Support|Nabriva Therapeutics AG: Grant/Research Support|Novartis Pharmaceuticals Corp.: Grant/Research Support|Paratek Pharmaceuticals, Inc.: Grant/Research Support|PureTech Health: Grant/Research Support|Sfunga Therapeutics: Grant/Research Support|Spero Therapeutics,: Grant/Research Support|Suzhou Sinovent Pharmaceuticals Co.: Grant/Research Support|TauRx Therapeutics: Grant/Research Support|Tetraphase Pharmaceuticals: Grant/Research Support|tranScrip Partners: Grant/Research Support|Utility Therapeutics: Grant/Research Support|Valanbio Therapeutics, Inc.: Grant/Research Support|VenatoRx: Grant/Research Support|Wockhardt Bio AG: Grant/Research Support.

